# A Systems-Oriented Multilevel Framework for Addressing Obesity in the 21st Century

**Published:** 2009-06-15

**Authors:** Terry T. Huang, Adam Drewnowski, Shiriki K. Kumanyika, Thomas A. Glass

**Affiliations:** Eunice Kennedy Shriver National Institute of Child Health and Human Development; University of Washington School of Public Health, Seattle, Washington; University of Pennsylvania School of Medicine, Philadelphia, Pennsylvania; The Johns Hopkins University Bloomberg School of Public Health, Baltimore, Maryland

Effective or sustainable prevention strategies for obesity, particularly in youths, have been elusive since the recognition of obesity as a major public health issue 2 decades ago. Although many advances have been made with regard to the basic biology of adiposity and behavioral modifications at the individual level, little success has been achieved in either preventing further weight gain or maintaining weight loss on a population level (1). To a great extent, this is the result of the complex task of trying to change the way people eat, move, and live, and sustaining those changes over time.

The most immediate cause of obesity is an imbalance of energy intake and energy expenditure in the body. This energy imbalance, on the magnitude seen in today's population, arises from the complex interactions of biological susceptibilities and socioenvironmental changes (2). Evidence in behavioral economics suggests that these powerful biological and contextual forces often place eating and exercise behavior beyond an individual's rational control (3). Therefore, the solution to the obesity epidemic lies in policies and interventions that alter those contextual features, taking individual biology and preferences into account. Historically, obesity research has been conducted within individual disciplines. Now, for both scientific inquiry and for public policies, obesity should be framed as a complex system in which behavior is affected by multiple individual-level factors and socioenvironmental factors (ie, factors related to the food, physical, cultural, or economic environment that enable or constrain human behavior, or both). These factors are heterogeneous and interdependent, and they interact dynamically (4).

Because of the complex system that affects obesity, researchers need to use a systems-oriented approach to address the multiple factors and levels. Whereas multidisciplinary research consists of teams with different expertise that can contribute to the understanding of particular aspects of a larger research question, truly cross-disciplinary research asks a priori questions and poses hypotheses that cut across disciplines and across levels of influence. For example, how do biological mechanisms of energy metabolism react to or how are they affected by different features of the built, social, or economic environment to produce a given distribution of eating or physical activity? How do these conditions enable or constrain eating and physical activity, and how are they embodied in biological systems to affect these behaviors?

In October 2007, the Eunice Kennedy Shriver National Institute of Child Health and Human Development (NICHD) convened the international conference Beyond Individual Behavior: Multidimensional Research in Obesity Linking Biology to Society. The goal was to create a climate of training, funding, and academic and institutional support for obesity research that will offer sustainable solutions to the obesity problem. Participants hoped to bridge the factors that influence obesity-related behaviors at the macro level (typically policies that shape and govern the food, physical, social, and economic environments in which we live) and the micro level (typically variables within people or their immediate surroundings that influence health outcomes). The conference was supported by the National Institutes of Health (National Cancer Institute; National Institute of Diabetes and Digestive and Kidney Diseases; National Heart, Lung, and Blood Institute; Division of Nutrition Research Coordination, Office of Behavioral and Social Sciences Research; and Office of Disease Prevention), the Canadian Institutes of Health Research (Institute of Nutrition, Metabolism, and Diabetes), and the Centers for Disease Control and Prevention. The content of this 3-day conference was designed to explicate the scientific foundation of this multilevel approach, generate research questions that apply to all disciplines, consider different intervention models, and discuss methods needed for the design and analysis of systems-oriented, multilevel studies (5). The essential elements of this multilevel agenda are framing obesity as a complex systems problem; encouraging cross-disciplinary questions and hypotheses; focusing on structural interventions (ie, modifications to the environment or policies); building capacity for multilevel research and action; and taking a global perspective.

## Theoretical Framework of the Multilevel Model to Address Obesity

**Figure 1:**
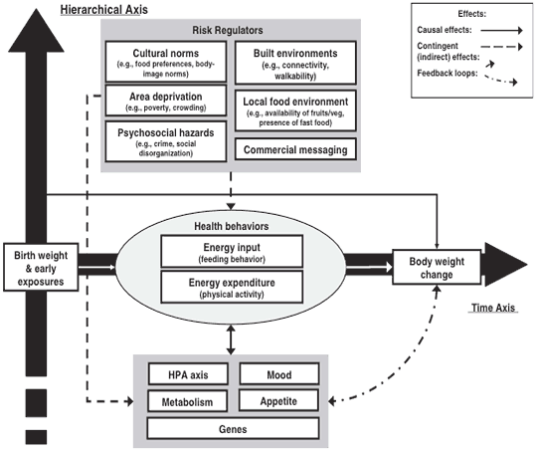
A systems-oriented, multilevel model applied to the study of obesity. The contingent effects of risk regulators (ie, embodiment, opportunity, and constraint) are shown with dotted arrows. "Causal" effects of biological and behavioral variables are shown with solid arrows. Feedback loops existing within grouped variables are not shown. Specific effects and multiple, time-ordered feedback loops between variables are not shown in order to reduce diagram complexity. Reprinted with permission from Elsevier (8).

Multilevel models are not new in public health; the concept stems from socioecological theories (6) that emphasize the importance of social and environmental factors in determining human behavior and health outcomes. However, the model has been interpreted to describe ecologic layers without elaborating on multiple sectors operating at multiple levels or including bidirectional interactions of factors (7). Glass and McAtee (8) present a multilevel model that is useful to address the complex, interacting contexts for obesity prevention. This model (Figure 1), which was a key focal point for the international conference, integrates biological (genes, cells, and organs) and socioenvironmental (economics, culture, social networks, and features of the physical environment) influences on behaviors such as eating and physical activity. Time, on the horizontal axis, is in the context of life course (conception to death) at the individual level or social change at the population level. The vertical axis depicts a nested hierarchy of systems including biological, social, and environmental influences (8). This model shows that the behaviors leading to health outcomes, not just health outcomes per se, are influenced by biological or socioenvironmental factors.

**Table:** 

The two main axes are the hierarchical axis, running from bottom to top, and the time axis, running from left to right. At the intersection of these two axes is birth weight and early exposures. From that point and moving to the right through time are health behaviors that include energy input (feeding behavior) and energy expenditure (physical activity). From these Health behaviors, continuing across through time, there is body weight change. A line indicates there is a causal connection between birth weight and early exposures and body weight change.
Connected to body weight change is a line showing a feedback loop back and forth to this group of factors: HPA axis, mood, metabolism, appetite, and genes; these also are connected back and forth with health behaviors as causal effects.
At the top of the hierarchical axis is a group of risk regulators: cultural norms (eg, food preferences, body-image norms), area deprivation (eg, poverty, crowding), psychosocial hazards (eg, crime, social disorganization), built environments (eg, connectivity, walkability), local food environment (eg, availability of fruits/veg, presence of fast food), and commercial messaging.
This group is connected to health behaviors by a contingent (indirect) effect and to the factors HPA axis, mood, metabolism, appetite, and genes.

The model is consistent with economics and psychology in that people are assumed to engage in behaviors based on preferences and attitudes. It becomes multilevel in that a person is constrained by factors that exert regulatory control on those behaviors. For example, food choices are made not just on the basis of preferences but also on the basis of the price of food, the cultural meaning of food, the availability of food, and the biological responses to the reward value of food. The distribution of these parameters constitutes a behavioral niche or landscape, to which the person must adapt and respond according to particular goals and intentions. The movement in time of higher or lower rates of obesity is, therefore, the result of multiply-dependent and interlocking systems. There are 4 possible implications. First, a single cause of the obesity epidemic is unlikely. Second, the processes that give rise to increasing average body size probably involve combinations of factors at multiple levels of influence. Third, small changes in 1 or more key factors may have large and potentially nonlinear influences on distribution of body weight. Finally, both socioenvironmental factors and biological processes are involved in the expression of human behavior.

One problem with building a systems-oriented, multilevel framework for obesity is that key influences in the physical or economic environment may not fit conventional definitions of causes. Glass and McAtee contend that social factors, such as social inequity and poverty, are difficult to study from a traditional epidemiologic standpoint, in part because they do not fit the definition of a causal risk factor (8). An alternative view of these variables is to define them as risk regulators, or dynamic components of interconnected systems that influence obesity-related behaviors from the personal level to the public policy level (8). Systems of food distribution alter the probabilities at a population level that these causes will align in ways that lead to different rates of obesity (9). The search for a set of key risk regulators provides greater room to consider the social, physical, cultural, and economic environments that influence obesity.

The concept of risk regulators also may help overcome some of the disadvantages of conventional socio-ecological models, namely the lack of clarity on what is most important, where the key drivers are located, or what the optimal intervention points are. The multiple levels (individual vs community) require a bridging structure, which act as conduits between macro-level forces and the factors in the local environment that govern eating and activity. The temporally and spatially distal forces that operate at the macro level cascade through organizations, through systems of food distribution, through policies and pricing, and eventually shape the reality that people perceive in their lives. Examples of the bridging in the case of obesity could be cultural norms, social networks, local food availability, food prices and taxes, physical activity amenities, psychosocial stress, or economic insecurity. These might act through neurologic or epigenetic  regulatory pathways to affect behavior and to generate feedback loops higher in the system. Epigenetic pathways are phenotypic differences between individuals that are not a result of genetic composition per se but a result of alterations in genetic expression through the silencing of genes or interference with genetic transcription.

## Forming Cross-Disciplinary Questions and Hypotheses for Research

**Figure 2:**
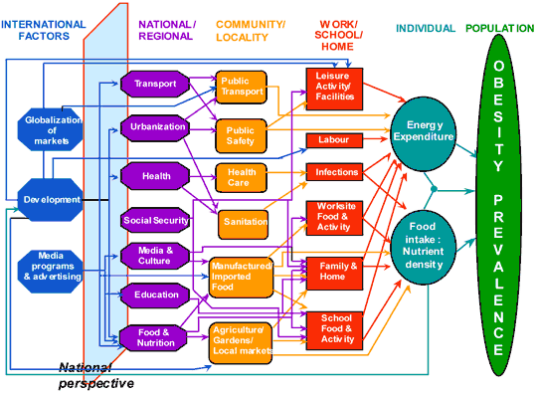
Prevalence (95% confidence interval) of obesity among children (2-19 years, age adjusted), according to family income as a percentage of the federal poverty level; the federal poverty level during 2004 was $18,850 for a family of 4 (7). Data source: National Health and Nutrition Examination Survey, 1999-2004.

Diverse sectors of society operate at different levels to influence population energy balance (Figure 2) (2,10). Factors can range from the individual level to the international level, and the sectors of influence include education, agriculture, transportation, urban developments, and media, among others, in addition to the health sector. Research that cuts across these different levels and sectors can be undertaken (Figure 2).

**Table:** 

This model shows the determinants that influence population energy balance at different levels. From left to right, they are International Factors, National/Regional, Community/Locality, Work/School/Home, and Individual, which lead to the population outcome Obesity Prevalence.
Within International Factors, the sectors are Globalization of markets, Development, and Media programs and advertising.
Globalization of markets is linked in International Factors to Development, in Community/Locality to Public Transport, and in Work/School/Home to Leisure Activity/Facilities. Development is linked in National/Regional to Urbanization, to Health, and to Food and Nutrition, in Community/Locality to Agriculture/Gardens/Local markets, and in Work/School/Home to Leisure Activity/Facilities and to Labour. Media programs and advertising is linked in National/Regional to Media and Culture, to Education, and to Food and Nutrition.
Between the International Factors and National/Regional is a marker denoting the National perspective that is also a factor in the model.
Within National/Regional, the sectors are Transport, Urbanization, Health, Social Security, Media and Culture, Education, and Food and Nutrition.
In National/Regional, the Transport sector and the Food and Nutrition sector are linked together, and the link between these two is linked in Community/Locality to Manufactured/Imported Food. The Urbanization sector, Health sector, and Food and Nutrition sector are linked together. Transport is linked in Community/Locality to Public Transport and to Public Safety. Urbanization is linked in Community/Locality to Public Transport, to Public Safety, and to Sanitation. Health is linked in Community/Locality to Health Care and to Sanitation. Social Security is linked in Work/School/Home to Leisure Activity/Facilities, to Family and Home, and to School Food and Activity. Media and Culture is linked in Community/Locality to Manufactured/Imported Food, and in Work/School/Home to Worksite Food and Activity and to Family and Home. Education is linked in Work/School/Home to Family and Home and to School Food and Activity. Food and Nutrition is linked in Community/Locality to Manufactured/Imported Food and to Agriculture/Gardens/Local markets, and is linked in Work/School/Home to Family and Home and to School Food and Activity.
Within Community/Locality, the sectors are Public Transport, Public Safety, Health Care, Sanitation, Manufactured/Imported Food, and Agriculture/Gardens/Local Markets.
Public Transport is linked in the Individual sector to Energy Expenditure. Public Safety is linked in Work/School/Home to Leisure Activity/Facilities and in the Individual sector to Energy Expenditure. Health care is linked in Work/School/Home to Infections. Sanitation is linked in Work/School/Home to Infections. Manufactured/Imported Food is linked in Work/School/Home to Worksite Food and Activity, to Family and Home, and to School Food and Activity, and in the Individual sector to Food intake: Nutrient density. Agriculture/Gardens/Local markets is linked in Work/School/Home to Family and Home and to School Food and Activity, and in the Individual sector to Food intake: Nutrient density.
Within Work/School/Home, the sectors are Leisure Activity/Facilities, Labour, Infections, Worksite Food and Activity, Family and Home, and School Food and Activity.
Leisure Activity/Facilities is linked in the Individual sector to Energy Expenditure. Labour is linked in the Individual sector to Energy Expenditure. Infections, Worksite Food and Activity, Family and Home, and School Food and Activity are each linked in the Individual sector to Energy Expenditure and to Food intake: Nutrient density.
Within Individual are 2 sectors, Energy Expenditure and Food intake: Nutrient density. Both are linked separately and together to the population outcome, Obesity Prevalence.

### Obesity as a function of biology

The simplest biological view of obesity is that energy intake (increased) and expenditure (decreased) became discordant over time. A decreased sensitivity to metabolic signals that inhibit overeating is highly adaptive for survival in circumstances where food availability is limited or cyclic, by permitting storage of excess energy, when available, as body fat. However, when an abundance of cheap, readily available, and palatable (eg, high-fat, high-sugar) food is in the environment, this raised threshold of metabolic tolerance promotes obesity (11). Failures in weight loss attempts are, in part, the result of powerful biological drives to store and maintain energy in the body.

An obesogenic prenatal environment can also increase the likelihood of obesity in the offspring through epigenetic effects (12). These epigenetic factors can be seen as biological risk regulators that might help explain, in part, how the environment is embodied in metabolic systems to affect behavior and health.

In animal studies, many prenatal manipulations appear to promote offspring obesity by permanently altering the development of central neural pathways that regulate food intake, energy expenditure, and energy storage (13). Human imaging studies suggest that the brain has automatic approach responses to food compared with nonfood objects (14) and that these responses can be influenced by product advertising (15) and pricing (16). The reward and executive control patterns in the brain can be induced and modulated by palatable, energy-dense foods in a way similar to addictive substances (17). These neural systems are powerful in defending the body from undernutrition but have little capacity to defend against overnutrition and upper limits of body weight and adiposity (18). So, what other factors in the environment trigger or alter people's biological response to food to make them eat in a way that promotes weight gain? Recent research points to elements of the social and physical environment, and emerging evidence also suggests that the economic and policy environment plays an important role. However, this area of research remains in its infancy. Furthermore, almost no research explores how macro-level variables influence biological processes to result in differential behavioral phenotypes or how biological drivers of obesity are affected by different socioenvironmental conditions.

Haemer et al (19) in this issue of *Preventing Chronic Disease*, explore in greater detail the biological risk regulators of obesity. In addition, Esposito et al (20) offer a developmental perspective to understanding how additional biopsychological factors interact with the family and school context to shape food preferences in children.

### Obesity as a function of the built environment

The availability, accessibility, and marketing of foods all contribute to our consumption patterns, either directly by enabling or constraining food choices or indirectly by modulating biological processes to affect eating. In the United States, the availability and accessibility of healthy foods, such as fresh produce, are often limited, particularly in poor or rural communities (21). Marketing of high-calorie foods via packaging, retail, and media to children has increased purchase and consumption of those foods (22).

Many features of the built physical environment may also affect energy expenditure. The lack of perceived safety, lack of facilities, and low access to key destinations (eg, inconvenient transportation) are some of the factors that inhibit or decrease physical activity levels (23). Physical activity improves insulin sensitivity, glucose homeostasis, and other metabolic profiles (24), which in turn can have an impact on adiposity (25). Reducing sedentary activity (eg, television viewing, computer usage) in children reduces obesity, but this effect appears to be mediated via a reduction in energy intake rather than an increase in physical activity (26). If so, neurologic responses may also act as mediators between sedentary activity and obesity.

With the emergence of geographic information systems technology, studying the built environment with objective measures in relation to obesity is now more feasible. Mechanisms of the association between the built environment and obesity remain poorly understood, particularly in terms of how the built environment interacts with biology to influence obesity-related behaviors. As Figure 1 illustrates, one should not assume that the relationships between environmental factors and health behavior are direct or linear.

### Obesity as a function of the social environment

Norms of food and physical activity behaviors and body image ideals vary by culture. Overweight in a child, for example, is viewed as a symbol of health by some cultures (27). In a simple computational experiment, Hammond (28) showed that changing norms of body weight, as the population becomes increasingly obese over time, could in themselves propagate obesity.

Cultural forces can also be barriers to obesity prevention. For instance, the American culture places a strong emphasis on individual responsibility over one's own lifestyle or the lifestyle of one's child. This cultural underpinning, in part, led to the conventional emphasis on research and programs to educate or train people how to behave in healthier ways. However, such individual-oriented approaches, which usually do not take into account biological and socioenvironmental drivers of behaviors, have rarely worked over the long term (29). Overcoming this fundamental aspect of our sociopolitical culture must be considered in a long-term solution to obesity.

Although many harmful social conditions (eg, poverty, pollution) can end lives prematurely, they are not susceptible to change by those most affected (ie, minority ethnic groups and children). Therefore, interventions that rely on individual health promotion alone will bias outcomes toward the more advantaged segments of the population, who have more choices about changing their environments. Examining health disparities through the lens of social disadvantage (eg, deprivation, discrimination-exclusion) rather than epidemiologic trends alone will influence research questions, comparisons, variables, and subgroups. Braveman discusses these concerns in this issue of *Preventing Chronic Disease *(30).

An increase in chronic stress (31) may be a way through which social conditions interact with biological processes to affect obesity-related behavior. Stress stimulates opioid release in the reward center of the brain, which is a defense mechanism for the body to attenuate the detrimental effects of stress. Chronic stress can repeatedly stimulate the reward pathways and further enhance the reward value of food, possibly contributing to increased energy intake and fat accumulation over time (32).

### Obesity as a function of economics

In the United States, data suggest that poverty is associated with higher obesity rates (33), whereas in many developing countries, higher rates of obesity are found in higher-income groups as a result of economic growth and improved standards of living (34). One explanation for these observations is that low-income groups in the United States and high-income groups in developing countries either are better able to afford or have greater access to energy-dense but nutrient-poor foods (35). These foods have high proportions of dietary fats, sugar, and refined grains, the cost of which has steadily decreased while the supply has steadily increased over the last 40 years (36). Nutrient-rich and energy-poor diets have much higher costs per calorie (37). Therefore, a testable hypothesis linking macro-level economics to obesity is that the higher cost of healthy foods may lead to financial stress. This, coupled with the higher availability, accessibility, and marketing of unhealthy foods in poorer neighborhoods, may lead to increased purchase and consumption of unhealthy foods, which over time results in increased obesity. Subsequently, increased obesity in the population can perpetuate itself through intergenerational epigenetic programming.

The food industry determines agricultural production, food manufacturing, processing, packaging, transport, retail, and marketing to influence the eating patterns of populations (38). The supply side of the food chain can be influenced by agricultural policies on farm output, while the demand side can be influenced by variables such as income, availability, and pricing. Furthermore, the foods that farmers choose to grow are influenced by policies that support some foods more than others; for example, corn and soybeans have, in general, more support than fruits and vegetables. It remains to be investigated how the different economic facets of food cause obesity variation across countries and people, how much can be attributed to the role of policy in affecting producer and consumer behavior, and how food production chains can be modified to shape future consumer demand for healthier food options.

## Structural Modifications to Multilevel Interventions

The next-generation interventions for obesity should start at the community level or higher, with multiple stakeholders that connect people, families, schools, government, and the private sector. Intervention activities should include not only educational schemes but also environmental changes to shift norms and enable the adoption of healthy behaviors within everyday life. The family, schools, primary care settings, and municipalities can be targeted simultaneously as catchment sites to interface with children and parents. Media organizations and businesses (eg, food manufacturers, retailers, supermarkets, the transportation industry) can also help shift norms, effectively contributing to both the supply and demand sides of the energy balance equation.

Much can be learned from the North Karelia Project in Finland from the 1970s through the 1990s, where that country's public health agency transformed the lifestyle pattern of Finnish communities to reduce smoking rates and improve dietary practices. The Finland project did not rely exclusively on individually focused educational interventions. The government created incentives for farmers to switch from meat to fruit and vegetable production, and worked through social networks by using community organizations. There were also efforts to use regulatory changes to influence the nutrient content of food (eg, requiring sausage makers to lower the fat content of their products across the entire market). The result was a greater than 50% reduction in coronary heart disease mortality, as well as reductions in stroke, cancer, and other diseases, in the entire country within 20 years (39).

Although research on multilevel interventions advances slowly, actions are already being taken in many US and international communities. This parallel movement at the grassroots level needs to be taken advantage of with rigorous evaluations to determine the effect of community-initiated interventions (40). There is little research on the dissemination and diffusion requirements of multilevel interventions. As intervention and evaluation research continue, dissemination must be part of the strategic effort.

## Capacity Building for Multilevel Research and Action

Multilevel research and interventions cannot be conducted or sustained if the agenda does not include a strong focus on building coalitions across societal sectors and increasing the capacity to tackle obesity (41). Specifically, public-private partnerships, leadership of national governments, and training of future multilevel researchers and policy makers are warranted.

### Public-private partnerships

Every person and every sector in society are important in a multilevel approach to obesity. The food industry and industries that shape our built environment have a role to play and should be invited to this research forum as partners (see Huang and Yaroch [42]). Industry not only shapes the physical landscape of our environment but also shapes values and norms. There is a need to agree on a public-private partnership framework that outlines the rules of these collaborations. Specifically, this framework must affirm that trade and health are not mutually exclusive. It should articulate issues related to trust-building, information sharing and technical cooperation, transparency of individual and collaborative efforts, and pooling of resources. Successful partnerships must be constructed through open, honest, and regular dialogues. As with any relationship, the partners must be willing to take risks and to compromise to find common ground. In addition, there must be leaders to champion the partnership and the cause the partnership represents. Finally, sufficient resources must be made available to implement any actions jointly developed by the partnership.

### Role of national governments

The experience in North Karelia and experiences in tackling tobacco use in the United States and other countries (43) suggest that top-down strategies must accompany bottom-up approaches to sustain the necessary environmental and behavioral changes to prevent obesity. Although individual-level interventions have been effective in reducing smoking, their effect never could have been sustained or scaled up to the population level in the absence of regulatory and economic interventions by the government (44). Many policy options have been proposed elsewhere (45), but few have been tested or evaluated to ascertain the evidence of cost-effectiveness.

National governments also play a critical role in facilitating and coordinating research and then translating research into programs and policies. Coordination is essential among government and nongovernment agencies as well as among different sectors in society. Leadership at the national level often is necessary to move a multilevel agenda forward. For example, since 2005 the Institute of Medicine has called for a national strategy on childhood obesity that cuts across government agencies and societal sectors (46), but such a national mandate has yet to be established (47).

### Training

Training of future scientists is an indispensable component of the long-term viability of any multilevel research agenda. Medical and public health training contain little to no curriculum on systems science. A coordinated effort is needed to develop training in a "multilevel science" in public health. Training should include not only the knowledge base of obesity and chronic disease prevention in general but also methodologic expertise for the design and analysis of multilevel studies, including novel statistical and computational approaches. Hammond discusses this training need in this issue (48). Training should be integrated at the predoctoral, postdoctoral, and midcareer levels.

## Obesity From a Global Perspective

In the world, approximately 22 million children younger than age 5 years are overweight. By 2015, an estimated 2.3 billion people aged 15 or older will be overweight and 700 million will be obese worldwide (49). By 2010, cardiovascular disease will be the leading cause of death in developing countries, and by 2030 more than 280 million people in developing countries will have type 2 diabetes (49). Key drivers of these numbers are transnational (globalization of markets and media, urbanization, trade, economic growth, food availability, marketing) (Figure 2), requiring a global perspective to address obesity. The increasing health effects related to obesity will pose substantial economic challenges as a result of cost and insufficient infrastructure in the world's health care systems (50). An unhealthy population leads to reduced economic productivity, which further exacerbates morbidity and mortality.

Experiences in the United States and other developed nations may serve as a starting point for understanding and combating obesity in developing countries. Nevertheless, factors may not all be equally relevant in different countries, and environmental, cultural, and sociopolitical influences within countries determine what types of solutions will be feasible and effective. More international research is needed to understand these differences. For example, the Seven Countries Study (51) on cardiovascular health provided great insight into the role of population-level variations in diet in heart disease risk. Although ecologic correlations are weak for supporting causal inference, this study was groundbreaking in showing population-level influences on disease rates and on preventive strategies. Obesity research can carry on these lessons. International research that capitalizes on the contrast on either differing obesity rates or differing socioenvironmental characteristics across contexts can be especially illuminating.

## Conclusions

Current levels of obesity reflect complex social changes and biological susceptibilities, and their interactions, during the last 40 years. Individual behaviors such as eating and physical activity do not occur in a vacuum; rather, they are influenced by socioenvironmental factors and by powerful biological processes. Behavior change cannot be sustained if these drivers of behavior are not considered. A systems-oriented, multilevel framework encompassing science and research capacity-building is the way to generate solutions that deal with the complex system in which obesity arises. A multilevel research agenda is cross-disciplinary, bringing together expertise in traditionally disparate fields to pose cross-disciplinary hypotheses and to test those hypotheses collectively. The agenda also would extend conventional research boundaries by tackling structural aspects of the social, physical, and policy environment that affect obesity. Capacity building for global research is critical for sustaining a multilevel research agenda for obesity and chronic disease prevention.

Ultimately, interventions should strive to make healthy eating and physical activity a natural and easy way of life. Using the framework discussed here, one approaches the problem by first looking at the whole picture rather than immediately zeroing in on a detail. Having a view, even if not a full understanding, of the relations among factors that regulate energy balance, across individuals as well as populations, allows one to simultaneously consider multiple leverage points in the system within which obesity occurs that can or need to be modified to yield the desired outcomes (52). Focused studies can then be designed to confirm and quantify these relationships and to test their effects. By nature, this systems-oriented, multilevel approach is solution-oriented, underlining the philosophy that mechanistic and intervention studies are worthy only if they can improve population health in a sustainable way. Given where we are today, faced with the continued lack of effective and sustainable prevention strategies, there is a critical need to implement this multilevel approach. We can do this by extending the boundaries of biomedical research to fill the gaps across all the disciplines relevant to obesity, from biological and behavioral sciences to social and policy research.
